# Low-dose adropin stimulates inflammasome activation of macrophage via mitochondrial ROS involved in colorectal cancer progression

**DOI:** 10.1186/s12885-023-11519-5

**Published:** 2023-10-30

**Authors:** Linghui Jia, Liting Liao, Yongshuai Jiang, Xiangyu Hu, Guotao Lu, Weiming Xiao, Weijuan Gong, Xiaoqin Jia

**Affiliations:** 1 Department of Basic Medicine, School of Medicine, Yangzhou University, Yangzhou, 225001 P. R. China; 2 Department of Gastroenterology, The Affiliated Hospital of Yangzhou University, Yangzhou, 225001 P. R. China; 3 Department of General Surgery, The Affiliated Hospital of Yangzhou University, Yangzhou, 225001 P. R. China; 4 Jiangsu Key Laboratory of Integrated Traditional Chinese and Western Medicine for Prevention and Treatment of Senile Diseases, Yangzhou, 225001 P. R. China; 5 Jiangsu Key Laboratory of Zoonosis, Yangzhou, 225001 P. R. China

**Keywords:** Adropin, Inflammasome, mROS, Macrophage, Colorectal cancer

## Abstract

Adropin is encoded by the energy homeostasis-associated (*ENHO*) gene and widely present in liver, pancreas, heart, kidney, brain, and vascular tissues. Abnormal adropin is associated with metabolic, inflammatory, immune, and central nervous disorders. Whether adropin is involved in the development of colorectal cancer (CRC) is still unclear. Here, decreased adropin expression of tumor-nest cells in advanced-stage CRC was demonstrated. Adropin expressed by carcinoma cells was negatively correlated with macrophage infiltration in the matrix of CRC tissues. However, tumor macrophages enhanced adropin expression and were positively correlated with tumor invasion and metastasis. *ENHO* gene transfection into colon cancer (MC38) cells inhibited tumor growth in vivo, accompanying the increase of M1 macrophages. Treatment with low-dose adropin (< 100 ng/mL) on macrophages ex vivo directly increased mitochondrial reactive oxygen species for inflammasome activation. Furthermore, ENHO^−/−^ mice had less M1 macrophages in vivo, and ENHO^−/−^ macrophages were inert to be induced into the M1 subset ex vivo. Finally, low-dose adropin promoted glucose utilization, and high-dose adropin enhanced the expression of CPT1α in macrophages. Therefore, variations of adropin level in carcinoma cells or macrophages in tumor tissues are differently involved in CRC progression. Low-dose adropin stimulates the antitumor activity of macrophages, but high-dose adropin facilitates the pro-tumor activity of macrophages. Increasing or decreasing the adropin level can inhibit tumor progression at different CRC stages.

**Supplementary Information:**

The online version contains supplementary material available at 10.1186/s12885-023-11519-5.

## Background

Adropin is a peptide hormone with 76 amino acids (MW: 4.499 kDa) encoded by the energy homeostasis-associated (*ENHO*) gene, including a secretory signal peptide (amino acids 1–33) and a biologically active fragment (amino acids 34–76). Humans, mice, and rats share the same amino acid sequence. Adropin plays a crucial role in modulating glucose and fatty acid metabolism [1, 2]. It also alleviates insulin resistance,[3] inhibits lipogenesis,[4] and improves endothelial cell function and neovascularization [5]. Clinical investigations demonstrated that decreased adropin level is comprehensively involved with metabolic diseases,[6] cardiovascular diseases, [7] and polycystic ovary syndrome [8].

The immunological effect of adropin has been identified in inflammation-associated diseases. Adropin could exert pro-inflammatory or anti-inflammatory effects by modulating the activities of macrophages, effectors, and regulatory T cells. Adropin decreases the expression of peroxisome proliferator-activated receptor γ (PPARγ) in adipose tissues and the liver to inhibit pre-adipocyte differentiation into mature adipocyte and thus reducing fat accumulation and macrophage infiltration-related inflammation [9]. On the contrary, adropin increases PPAR-γ expression in macrophages to induce M2 polarization,[10] which depends on the energy provided by fatty acid oxidation [11, 12]. Tumor-associated macrophages (TAM), which account for 30–70% of tumor interstitial cells also utilize energy from fatty acid oxidation [13]. However, how TAM activities are regulated by adropin remains unknown.

Colorectal cancer (CRC) is the third popular type of cancer worldwide. Most core metabolic pathways, including glucose, glutamine, amino acid, and lipid metabolism, substantially vary in CRC cells [14, 15]. Metabolic reprogramming by cancer cells would be exploited to provide more energy and materials for rapid cell division [16]. The involvement of adropin and its putative receptor, G-protein coupled receptor 19 (GPR19), on tumor cell is only studied in human breast carcinoma. GPR19 overexpression drives MDA-MB-231 cells towards an epithelial phenotype. Adropin’s action on GPR19 initiates MET in mesenchymal like breast cancer cells [17]. In the present study, we aimed to clarify how variations in adropin affect CRC progression and whether adropin exerts modulatory effects on macrophages involved in tumor progression.

## Mehods

### Bioinformatics analysis

Based on The Cancer Genome Atlas (TCGA, https://portal.Gdc.Cancer.gov), samples of 51 normal individuals and 646 tumor patients were collected for the analyses of *ENHO* and *GPR19* gene expression (level 3 HTSeq-FPKM), immune system infiltrates, and related clinical information. All patients with CRC (*n* = 644) were divided into two groups according to adropin level and GPR19 expression. R language (version 3.6.3) was used to analyze ENHO and GPR19 mRNA data and clinical features. TNM stage was classified following the 8th edition (2017) of TNM Classification for CRC by the American Joint Committee on Cancer. The survival of patients with CRC with different adropin and GPR19 expression was analyzed by Kaplan–Meier (K-M) survival analysis.

### Patients and biopsies

Tumor biopsies of 68 patients with CRC (43 males and 25 females) aged 31–82 years old with an average age of 62.7 years were collected from the Affiliated Hospital of Yangzhou University. Normal issues surrounding the tumor tissues (5 cm away from tumor tissues) were collected as the control. All tumor tissues were confirmed as adenocarcinoma by two independent pathologists. The clinical features of the patients are listed in Supplementary Table 1. Four patients with CRC and liver or lung metastasis (Supplementary Table 2) were recruited for the comparison of adropin and GPR19 expression between primary and metastatic tissues. The Ethics Committee of the Affiliated Hospital of Yangzhou University approved this study. All patients signed informed consent forms.

### Reagents, animals, and antibodies

A lentivirus vector system was purchased from GeneChem (Shanghai, China). Recombinant adropin was purchased from Signalway Antibody (AP82783, Maryland, USA). Recombinant IFN-γ was from Absin (abs04127, China), TGF-β1 (7666-MB-005) was from R&D Systems (California, USA), IL-4 (HY-P70653) and IL-10 (HY-P70517) were from MedChem Express (New Jersey, USA), and LPS (L2654) was from from Merck. ENHO-deficient mice with C57BL/6 background were generated in GemPharmatech (Nanjing, Jiangsu Province, China) and routinely bred in the Center for Comparative Medicine of Yangzhou University. All animal experiments were approved by the Institutional Animal Care and Use Committee of Yangzhou University (Yangzhou, China).

The antibodies used for immunohistochemistry were as follows. Adropin (ab224725) was from Abcam (Cambridge, UK); GPR19 (Orb161223) was from Biorbyt (Cambridge, UK); CD68 (66231-2-IG), arginase 1 (ARG1, 66129-1-IG), and PD-L1 (66248-1-IG) were from Proteintech (Wuhan, China); and antibody (MA5-17139) against inducible nitric oxide synthase (iNOS) was from Invitrogen (California, USA).

The antibodies for Western blot were as follows. Adropin (NBP1-26387) was from Novus (Colorado, USA). NLRP3 (D4D8T), caspase-1 (E2Z1C), IL-1β (D3U3E), HK2 (C64G5), C/EBPβ (D56F10), and actin (3H6G5) were from Cell Signaling Technology (Boston, MA, USA). ARG1 (66129-1-IG), iNOS (MA5-17139), and GLUT1 (66290-1-IG) were from Proteintech (Wuhan,China). CPT1α (EPR218–43-71-2 F) and PPARγ (Ab23673) were from Abcam (Cambridge, UK).

The following antibodies were used for flow cytometry and sourced from eBioscience, Biolegend, and BD Pharmingen: CD11b (M1/70), F4/80 (BM8 or T45-2342), CD16/32 (B335358), CD206 (C068C2), CD86 (GL-1), CD80 (11-0801-82), CD3 (145-2C11), CD8a (53 − 6.7), and IL-1β (12-7114-82).

### Immunohistochemistry and histological evaluation

All biopsies were fixed in 10% neutral buffer formalin overnight, embedded in paraffin, and cut into sections. After antigenic epitopes were retrieved, the sections were routinely incubated with primary antibody overnight and stained with peroxidase-conjugated or fluorescein-conjugated secondary antibody. Finally, the sections were stained, observed under a high-power optical microscope, and evaluated [18].

### Isolation, culture and treatment of macrophages from spleen

Spleen single-cell suspension were obtained by grind, cracking and centrifugation.

Splenic macrophages were isolated from spleen single-cell suspension using Anti-F4/80 MicroBeads UltraPure and then cultured in complete culture medium (RPMI 1640 containing 10% exosome-free FBS, supplemented with 50 mg/ml penicillin/streptomycin) in a humidified incubator with 5% CO2 at 37 °C. The macrophages were polarized into M1 in the presence of 100ng/mL LPS and 20ng/mL IFN-γ(20ng/mL) for 24 h,or into M2 by adding IL-4(50ng/mL) or IL-10(50ng/mL) or TGF-β(25ng/mL) for 24 h. Splenic macrophages were treated with Adropin(10,30,100,200,400ng/mL) for 24 h.

### Tumor transplantation

MC38 cells were acquired from American Type Culture Collection.MC38 cells were transduced with the indicated ENHO-recombinant lentivirus. Then, MC38 cells with stable adropin expression were selected with puromycin (2 µg/mL). Logarithmic-phase MC38 cells (1.5 × 10^6^) were subcutaneously injected into the left dorsal part of 8-week-old male *C57BL*/6 mice. Tumor diameters were monitored every 3 days. On day 20, the mice were euthanized, and tumor tissues were dissected from the mice. The tumor tissues were cut into small pieces and ground. Mononuclear cells were passed through a filter net and enriched by density-gradient centrifugation with 30% percoll solution for further analysis.

### Flow cytometry

Mononuclear cells were stained with corresponding antibodies, detected by BD FACS Verse, and analyzed by FlowJo software. For intracellular staining, macrophages were fixed, permeabilized, stained with the antibody against IL-1β, and analyzed by flow cytometry. DCFH-DA (Beyotime, S0033M) and MitoSOX Indicator (Thermo Fisher Scientific, M36008) were used to evaluate the levels of cytoplasmic reactive oxygen species (cROS) and mitochondrial reactive oxygen species (mROS).

### Western blot

Cell lysates were generated, separated by polyacrylamide gel electrophoresis, and transferred to polyvinylidene fluoride membrane. Then, the membranes were stained with primary and secondary antibodies. Imprinted signals were developed with an enhanced chemiluminiscent kit (Vazyme).

### Statistics

Differences between two groups were analyzed by grouped student *t* test, and comparisons of more than two groups of data were analyzed by ANOVA. Spearman correlation coefficient was used to analyze the linear correlation. All analyses were carried out using GraphPad Prism 9.0. Significance of difference was indicated by *P* < 0.05 (*), *P* < 0.01 (**), and *P* < 0.001 (***).

## Results

### Decreased adropin expression of nest cells in CRC tissues

**Fig. 1:**
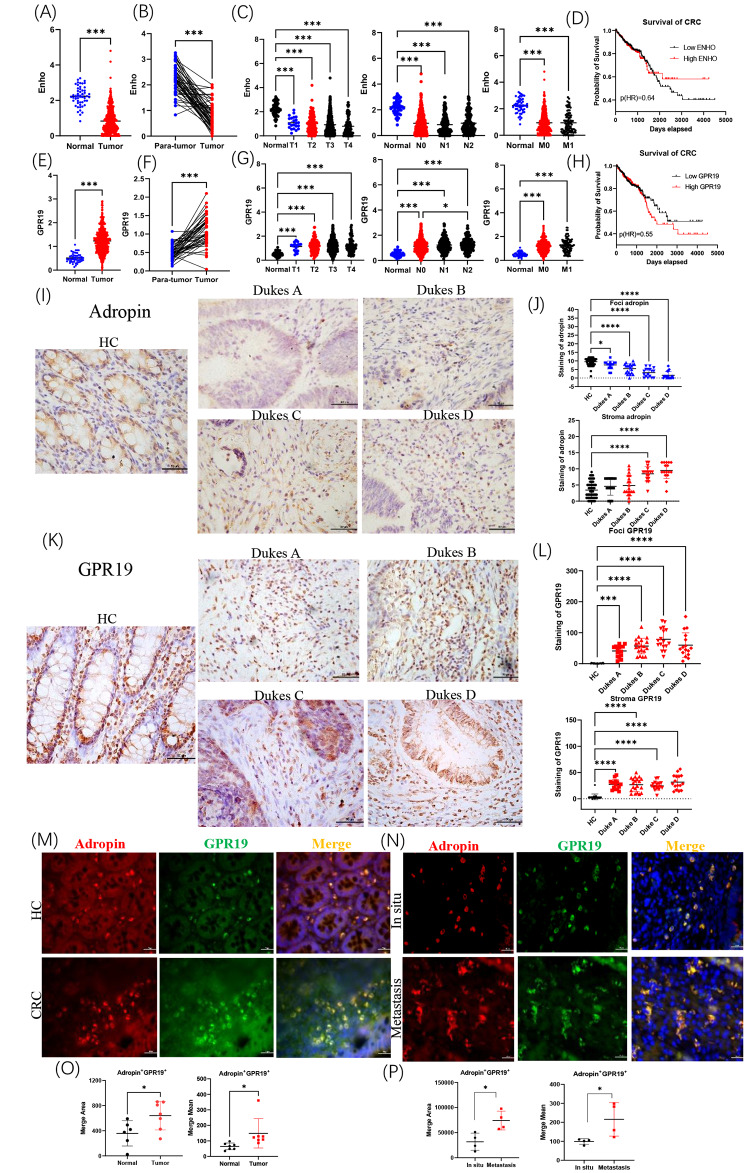
Variations of adropin expression in CRC biopsies. Comparison of ENHO (**A**) and GPR19 transcriptions (**E**) between tumor and normal tissues. Comparison of ENHO (**B**) and GPR19 transcriptions (F) between tumor tissues and matched surrounding tissues. Variations of ENHO (**C**) and GPR19 transcriptions (**G**) in CRC under different TNM stages. Survival analysis of patients with high or low ENHO (**D**) or GPR19 (**H**). Histological analysis of adropin (**I, J**) or GPR19 (**K, L**) in CRC biopsies. Double staining of adropin and GPR19 in normal, in situ (**M**), and metastatic tumor (**N**) tissues. Comparing positive area and mean fluorescent intensity of double-positive cells (**O, P**). *, *P* < 0.05; **, *P* < 0.01; ***, *P* < 0.001

Based on the analysis by TCGA, ENHO mRNA level substantially decreased in tumor tissues (Fig. 1A) compared with normal colon tissues. The transcriptional level of ENHO was lower in tumor tissues than in the surrounding normal tissues (Fig. 1B). Despite the low transcription level of ENHO in patients with CRC, no differences in ENHO levels were found among the patients at different TNM stages (Fig. 1C). Moreover, the survival of patients with high ENHO was prolonged but without statistical difference (Fig. 1D). GPR19, as an adropin receptor, is widely expressed by tumor cells [19, 20]. Elevated GPR19 transcription in CRC tissues was observed (Fig. 1E), and GPR9 had higher transcription in tumors than in para-tumor tissues (Fig. 1F). Although patients in T1–T4 stages had increased GPR19 transcription, no differences in GPR19 mRNA levels were found among the patients at different TNM stages (Fig. 1G). We did not observe statistical differences in the survival of patients with high and low GPR19 level (Fig. 1H). Collectively, these results indicated that adropin and GPR19 have a role in CRC development.

Adropin and GPR19 protein expression in CRC biopsies was further analyzed by histochemistry. The presence of adropin or GPR19 in the nest or matrix cells of CRC tissues was separately examined (Fig. 1I K). The adropin level of tumor foci decreased, which is consistent with the bioinformatics analysis. Unexpectedly, elevated adropin expression in stroma cells was seen in advanced CRC stages (Dukes C-D, Fig. 1J), indicating that adropin would be used by tumor matrix cells to promote cancer progression. The presence of GPR19 in the nest or matrix cells of CRC biopsies also increased (Fig. 1L). Furthermore, immunofluorescence revealed that more adropin^+^ GPR19^+^ cells were present in the stroma of CRC biopsies than in normal tissues (Fig. 1M and O, and Supplementary Fig. 1) and more adropin^+^ GPR19^+^ cells were present in the stroma of metastatic CRC biopsies compared with in situ tumors (Fig. 1 N and 1P). In conclusion, adropin/GPR19 is involved in CRC progression, and variations of adropin expression in tumor nest or stroma cells have differently clinic relevance.

### Adropin in tumor nest or matrix cells correlated differently with macrophage infiltration

**Fig. 2:**
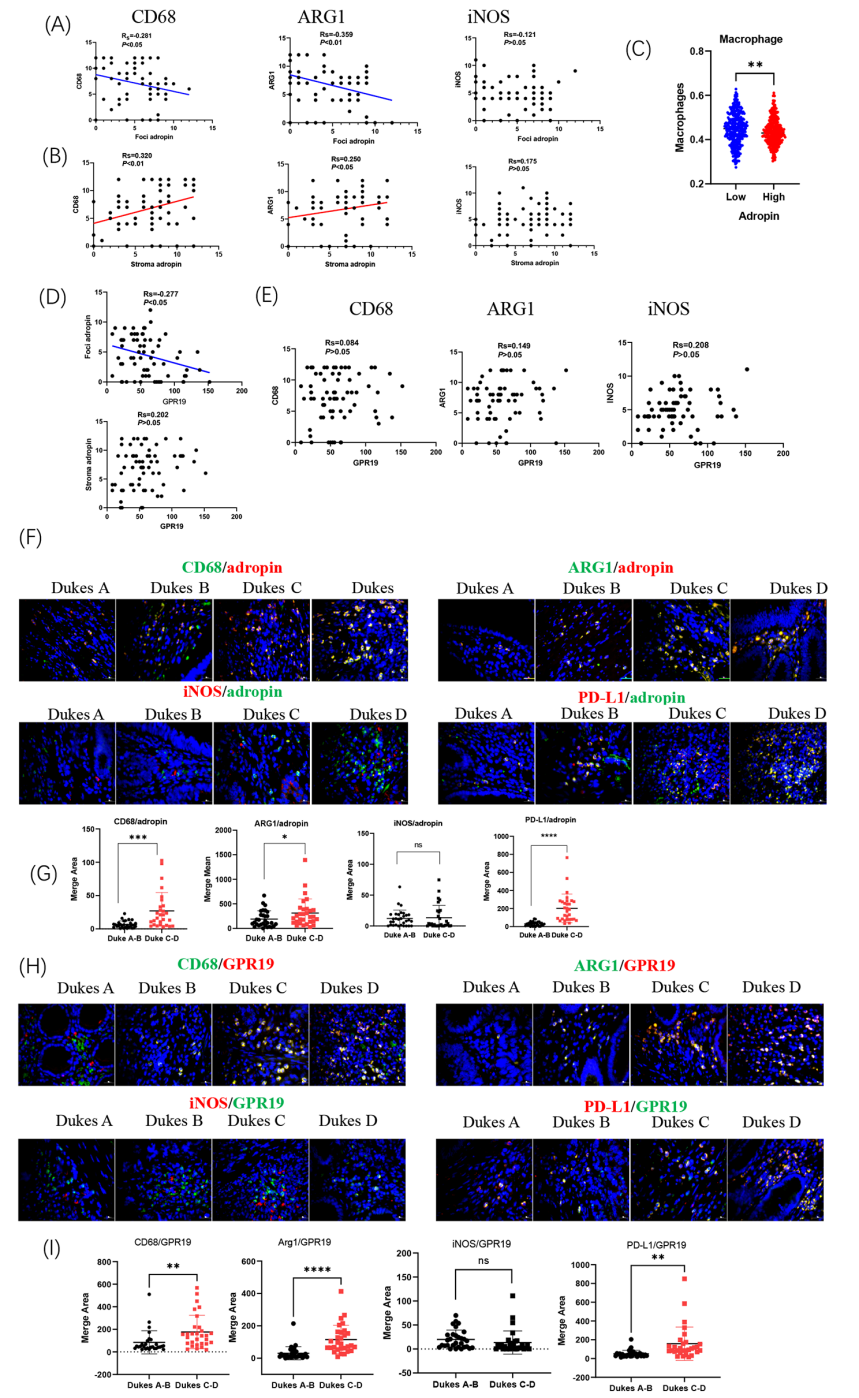
Correlational analysis of adropin level with local macrophages in CRC. Correlation study of adropin with CD68, ARG1, or iNOS in tumor foci (**A**) or stromal cells (**B**). (**C**) Comparison of infiltrated macrophages between high and low ENHO transcriptions. (**D**) Correlation study of GPR19 with adropin in foci and stroma. (**E**) Correlation study of GPR19 with CD68, ARG1, or iNOS. Double staining of adropin and CD68, ARG1, iNOS, or PD-L1 (**F**) and statistics (**G**). Double staining of GPR19 and CD68, ARG1, iNOS, or PD-L1 (**H**) and statistics (**I**). ns, no significance; *, *P* < 0.05; **, *P* < 0.01; ***, *P* < 0.001

We investigated how the adropin produced by tumor nest cells affected macrophage infiltration. The results showed that the staining intensity of adropin in tumor foci was negatively correlated with local CD68 and ARG1, which is produced by M2 macrophage, but had no associations with iNOS, which is a marker of M1 macrophage (Fig. 2A). However, adropin in tumor stroma cells was positively correlated with local CD68 and ARG1 without any effects on iNOS (Fig. 2B). As confirmed by TCGA analysis, the CRC biopsies with high adropin transcription had less macrophage infiltration (Fig. 2C). GPR19 in CRC cells was negatively correlated with adropin in the tumor foci but had no relationships with adropin in the tumor stroma (Fig. 2D). Moreover, GPR19 had no associations with the staining of CD68, ARG1, or iNOS (Fig. 2E).

Given macrophages are widely distributed in the matrix of tumor tissues [21], adropin expression in the TAMs of CRC biopsies was further checked. As shown in Fig. 2F, more adropin^+^ CD68^+^, adropin^+^ ARG1^+^, and adropin^+^ PD-L1^+^ cells were seen in the advanced stages of CRC (Dukes C-D), but no variations in adropin^+^ iNOS^+^ cells were observed between early-stage (Dukes A-B) and late-stage CRC (Dukes C-D)(Fig. 2F and G). In parallel, GPR19^+^ CD68^+^, GPR19^+^ ARG1^+^, and GPR19^+^ PD-L1^+^ cells increased in late-stage CRC, whereas GPR19^+^ iNOS^+^ cells had no variation (Fig. 2H and I). Together, the results showed that adropin in tumor nest cells was negatively associated with M2 macrophages, but adropin in tumor stroma cells was positively correlated with more M2 macrophage infiltration and facilitated tumor evasion.

### Ectopic expression of adropin suppressed tumor growth in vivo

**Fig. 3:**
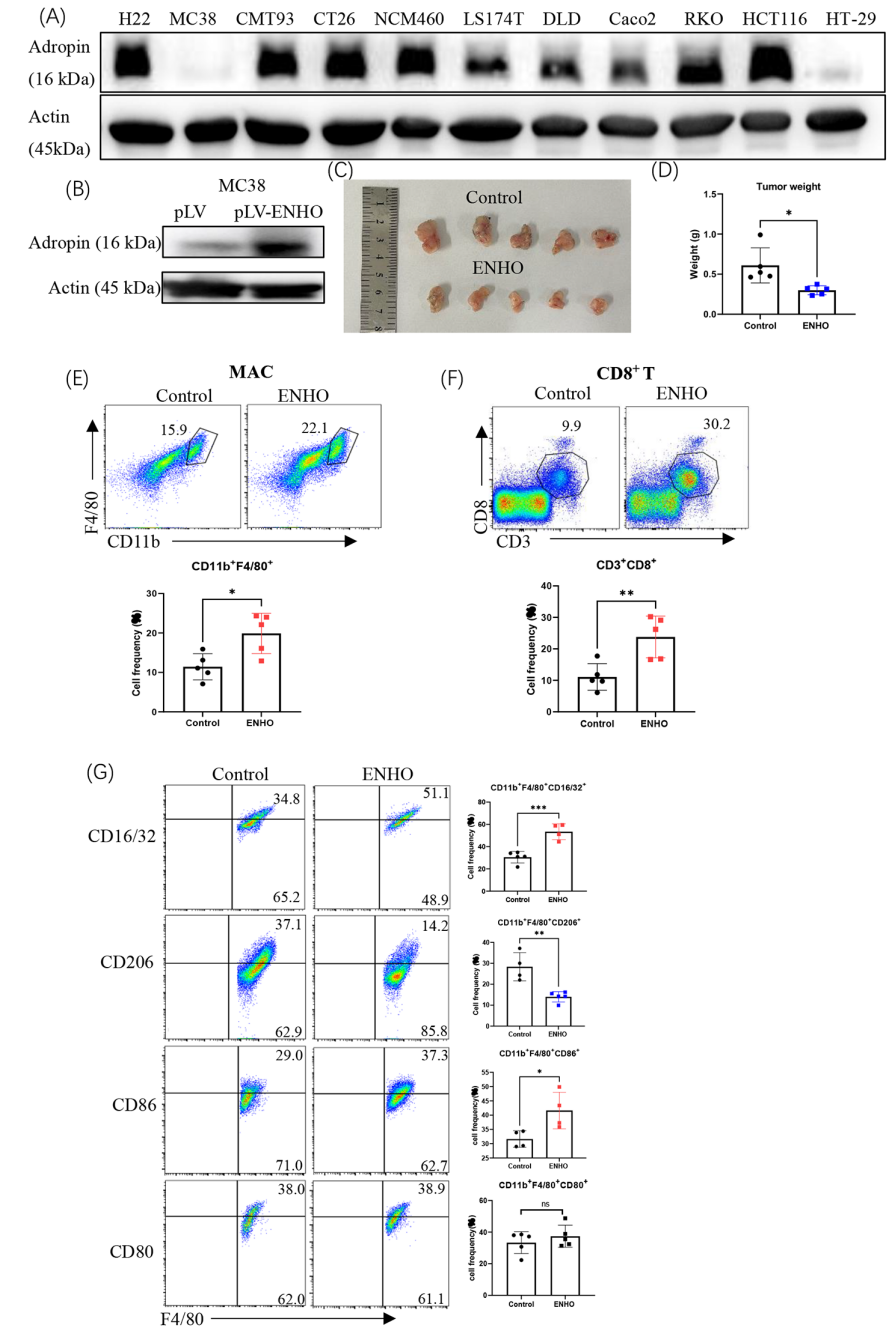
Ectopic adropin expression inhibits tumor growth in vivo. (**A**) Presence of adropin in the indicated cell lines. (**B**) Ectopic adropin expression in MC38 cells by lentivirus transfection. (**C**) In vivo tumors formed by MC38 and MC38-ENHO cells. (**D**) Weights of tumors. Detection of macrophages (**E**) and CD8^+^ T cells (**F**) in tumors. (**G**) Phenotypic analysis of tumor-infiltrated macrophages. The experiment was repeated twice. ns, no significance; *, *P* < 0.05; **, *P* < 0.01; ***, *P* < 0.001

We aimed to elucidate how the adropin produced by tumor cells affects tumor progression. A variety of alimentary tumor cell lines were used to analyze adropin expression. We found that MC38 cells had adropin deficiency (Fig. 3A). Adropin was ectopically expressed by MC38 cells transfected with an ENHO-recombinant lentivirus (Fig. 3B). When MC38 or MC38–adropin cells were subcutaneously injected into mice, the tumors had smaller size (Fig. 3C) and lesser weight (Fig. 3D) in the mice with MC38-adropin cells. More macrophages (Fig. 3E) and CD8^+^ T cells (Fig. 3F) were also present in MC38–adropin-transplanted tumor tissues. The MC38–adropin tumors had more CD16/32^+^ or CD86^+^ macrophages but had less CD206^+^ macrophages. We did not observe any changes in CD80 in these macrophages (Fig. 3G). Therefore, ectopic adropin expression in tumor cells exerts antitumor effects involving less M2 macrophages and more M1 cells.

### Low-dose adropin stimulated inflammasome activation ex vivo

**Fig. 4:**
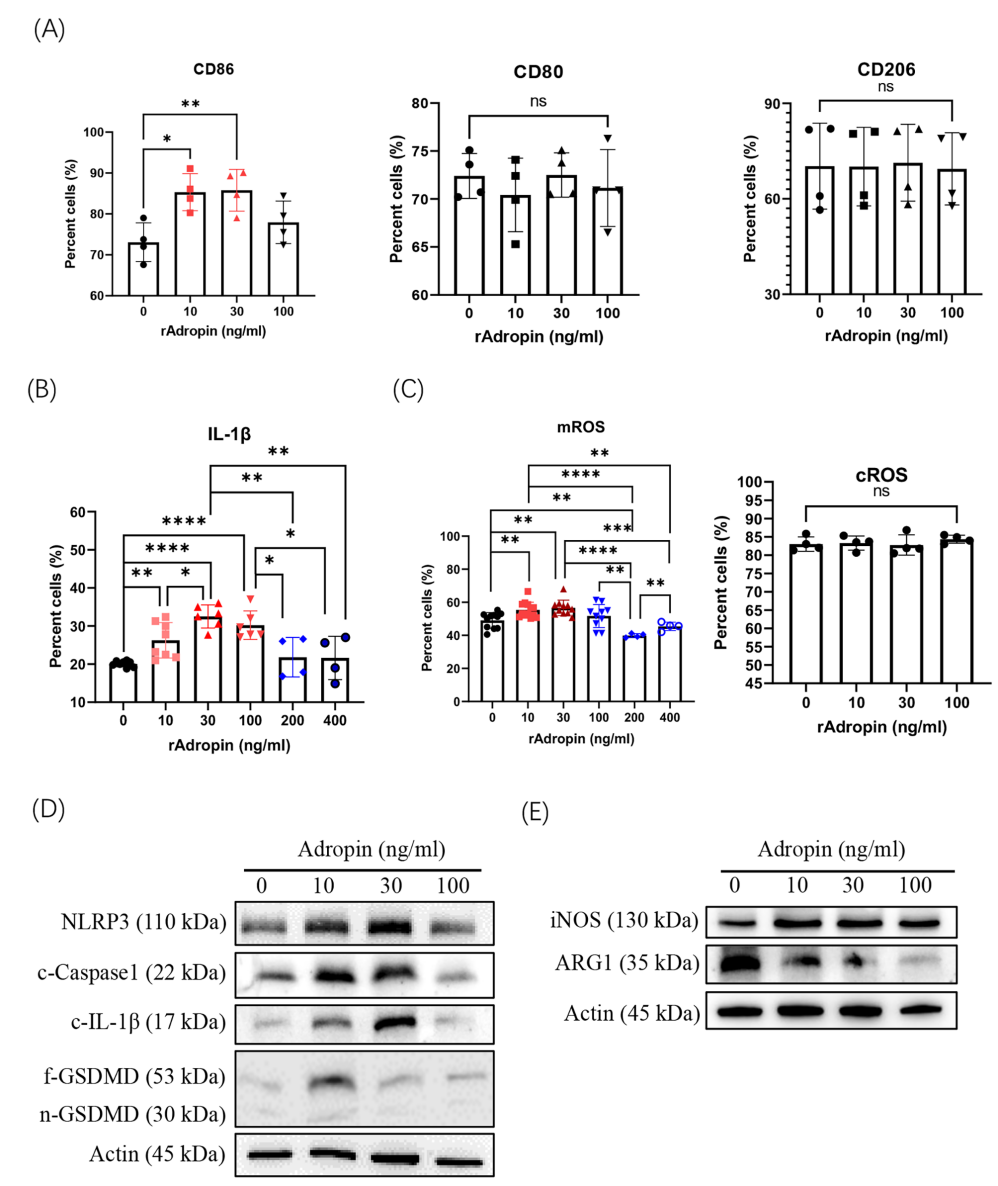
Effects of recombinant adropin on macrophage activities ex vivo. (**A**) Variations of CD86, CD80, and CD206 on macrophages treated by recombinant adropin. Production of IL-1β (**B**), mROS, and cROS (**C**) detected by flow cytometry. Inflammasome-associated molecules (**D**) and effector molecules (**E**) of macrophages checked by Western blot. The experiment was carried-out three times. ns, no significance; *, *P* < 0.05; **, *P* < 0.01; ***, *P* < 0.001

The macrophages were treated with various doses of recombinant adropin ex vivo. Low-dose adropin (10 and 30 ng/mL) stimulated CD86 expression but had no obvious effects on CD80 and CD206 macrophages (Fig. 4A, Supplementary Fig. 1). IL-1β production was also increased by adropin stimulation at the doses of 10 and 30 ng/mL (Fig. 4B, Supplementary Fig. 2). Considering that adropin promotes glucose oxidation in cells,[22] mROS and cROS in adropin-treated macrophages were determined. Despite no obvious changes in total intracellular ROS were observed, mROS increased in macrophages in a dose-dependent manner (Fig. 4C, Supplementary Fig. 2). mROS is able to stimulate NLPR3 activation;[23, 24] thus, NLRP3 and cleaved caspase1 (c-Caspase 1) were increased in macrophages by adropin (10–30 ng/mL). As a result of inflammasome activation, cleaved IL-1β (c-IL-1β) and the N-terminal fragment of gasdermin D (n-GSDMD) were also elevated (Fig. 4D). Increased iNOS and decreased ARG1 were found in the adropin-treated macrophages (Fig. 4E). Apparently, high-dose adropin (100 ng/mL) inhibited the inflammasome activation of macrophages (Fig. 4D). When macrophages were treated by higher adropin dose (200 and 400 ng/mL), IL-1β and mROS were decreased (Fig. 4B and C,supplementary Fig. 3), indicating that the stimulatory effect of adropin on macrophage only works at low dose (< 100 ng/mL).

### ENHO-deficient mice had less M1 macrophages

**Fig. 5:**
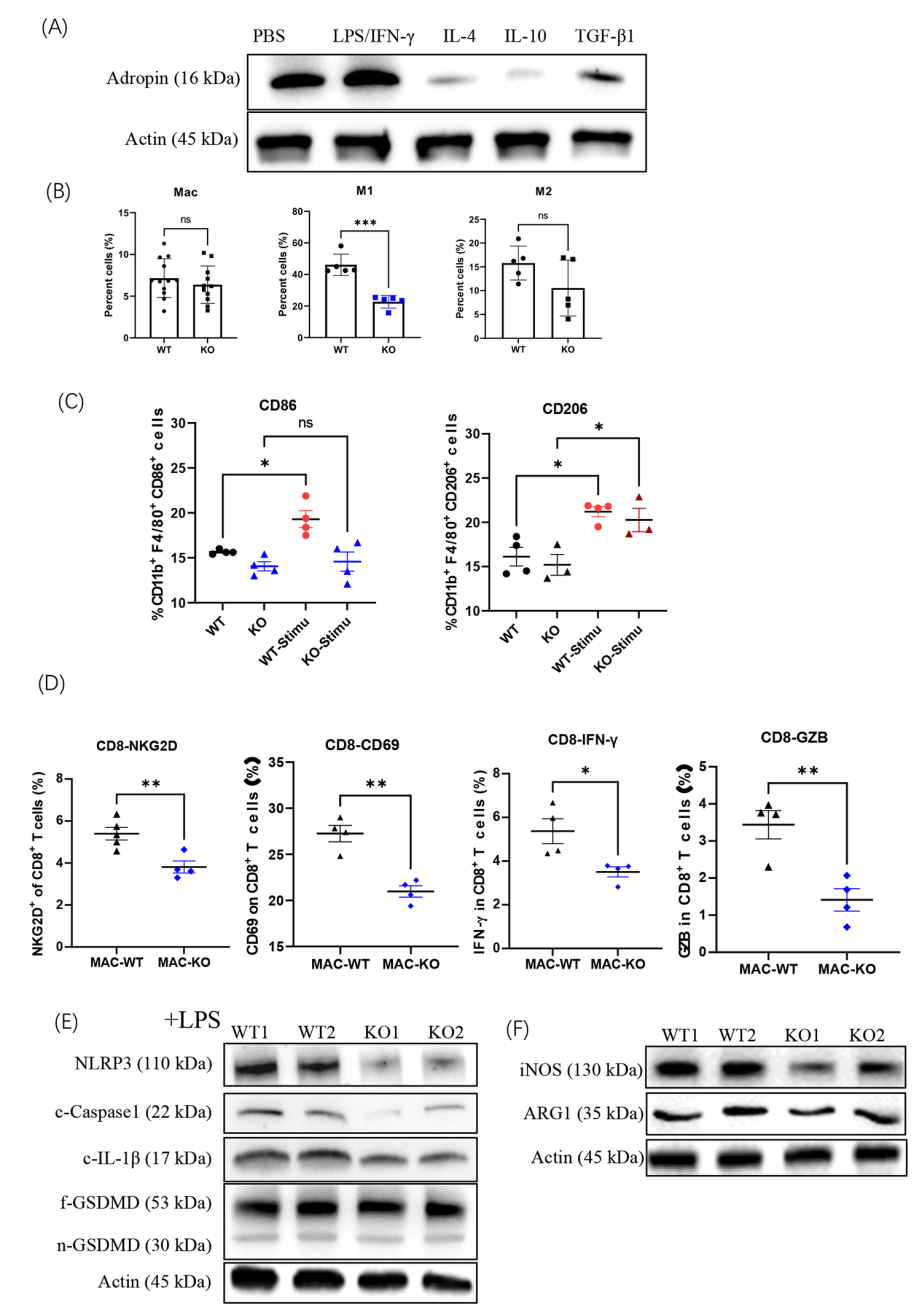
Macrophages in *ENHO*^**−/−**^ mice. (**A**) Adropin variations in macrophages after the treatment of LPS/IFNγ, IL-4, IL-10, or TGF-β1. (**B**) Macrophage subsets in ENHO^−/−^ mice. (**C**) CD86 and CD206 expression on WT or KO macrophages treated by LPS/IFNγ or IL-4. (**D**) Inhibitory effects of WT or KO macrophages on CD8^+^ T cells. Inflammasome-associated molecules (**E**) and effector molecules (**F**) of WT or KO macrophages. The experiment was carried-out three times. ns, no significance; *, *P* < 0.05; **, *P* < 0.01; ***, *P* < 0.001

Next, we checked the intrinsic secretion of adropin by M1 or M2 macrophages. M1 macrophages had increased adropin production as stimulated by LPS/IFN-γ, but M2 macrophages had decreased adropin production after being treated by IL-4, IL-10, or TGF-β1 (Fig. 5A). Furthermore, although no substantial changes in total macrophages were found in the spleens of ENHO-deficient mice (Supplementary Fig. 4A), the M1 population had less frequency, whereas the M2 subset had no changes (Fig. 5B, Supplementary Fig. 4B). When macrophages of wild-type (WT) or knout-out (KO) mice were treated with LPS/IFN-γ ex vivo, less CD86^+^ macrophages were induced in the KO mice compared with the WT mice. Macrophages in the two groups were treated with IL-4 and showed comparable CD206 expression (Fig. 5C, Supplementary Fig. 4C). These ENHO^−/−^ macrophages exerted less stimulatory effects on the NKG2D, CD69, IFN-γ, and granzyme B expression of CD8^+^ T cells (Fig. 5D, Supplementary Fig. 4D), confirming that ENHO-deficient macrophages comprised M1-like activities. Expression levels of NLRP3, c-caspase1, and c-IL-1β also decreased in ENHO^−/−^ macrophages (Fig. 5E). No variations in GSDMD activation were observed possibly because of the low baseline activation in the two macrophages. Accordingly, adropin-deficient macrophages had decreased iNOS production, whereas their ARG1 expression did not change (Fig. 5F). These results demonstrated that adropin-deficient mice had less M1 macrophages in vivo.

### Pro-inflammasome effect of adropin depends on mROS

**Fig. 6:**
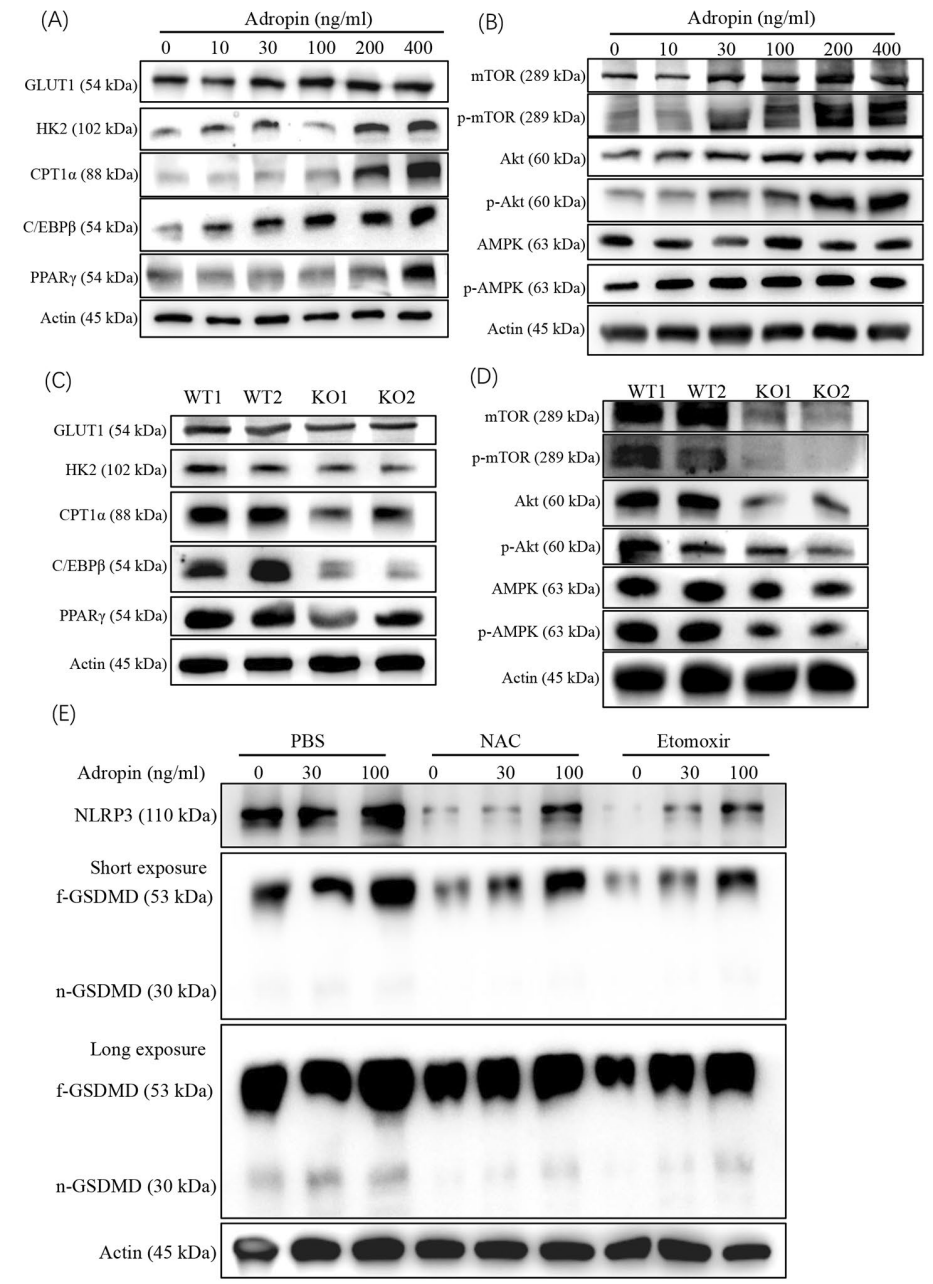
Metabolic molecules in macrophage with adropin treatment. (**A**) Variations of key molecules involved in glycol–lipid metabolism. (**B**) Akt, mTOR, and AMPK levels of adropin-treated macrophages. (**C**) Detection of the above metabolism molecules in ENHO^−/−^ macrophages. (**D**) Akt, mTOR, and AMPK levels of ENHO^−/−^ macrophages. (**E**) Effect of ROS depletion or CPT1α inhibition on inflammasome activation. Each experiment was repeated at least twice

Given that adropin promotes glucose oxidation via activating pyruvate dehydrogenase (PDH) and downregulating PDH kinase-4 that inhibits PDH,[25] several restrictive enzymes in glucose and fatty acid catabolism were checked in the macrophages after adropin treatment. Glucose transporter 1 (GLUT1), which indicates glucose uptake, and hexokinase 2 (HK2), which indicates glucose utilization, were upregulated in macrophages at low-dose adropin (< 100 ng/mL). In comparison, carnitine palmitoyl transferase 1 (CPT1α), which indicates fatty acid oxidation, was only upregulated at high doses of adropin (> 100 ng/mL). The CCAAT enhancer binding protein (c/EBPβ) as a key nuclear factor for modulating energy genesis [26] was increased by adropin in a dose-dependent manner. Moreover, PPARγ was only upregulated under the stimulation of high adropin concentration (Fig. 6A).

Mammalian target of rapamycin (mTOR), as a key regulator for cell proliferation, activation, and glucose metabolism, is at the crossroads of the PI3K/Akt and AMPK signaling pathways [27]. Phosphorylated mTOR and Akt were increased in macrophages by adropin treatment, whereas AMPK had no changes (Fig. 6B). GLUT1, HK2 and CPT1α decreased with low c/EBPβ and PPARγ levels in ENHO^−/−^ macrophages (Fig. 6C). In addition, mTOR, Akt. and AMPK decreased in ENHO^−/−^ macrophages, reflecting the effects of the persistent absence of adropin (Fig. 6D) in vivo. Finally, when adropin-stimulated macrophages were depleted of cellular ROS by N-acetyl-L-cysteine or CPT1α inhibitor (Etomoxir), the expression of NLRP3 and n-GSDMD, was remarkably reduced (Fig. 6E). The results confirmed that adropin promotes oxidative reaction in the mitochondria at a low dose to generate mROS for inflammasome activation.

## Discussion

A growing body of literature has suggested a link between obesity and cancer [28]. Adropin as an energy-homeostasis protein would affect the activities of tumor cells or immune cells in the matrix. Here, tumor nest cells decreased adropin expression, but matrix cells, particularly macrophages, increased adropin production in advanced CRC stages. The high adropin level in nest cells indicated less TAM infiltration, but increased adropin in matrix cells was correlated with more TAMs in CRC with tumor invasion and metastasis. When MC38 cells with ectopic adropin expression were transplanted into mice, tumor growth was inhibited accompanying an increase in M1 macrophages in the tumor microenvironment. Low-dose recombinant adropin (< 100 ng/mL) could directly stimulate the M1-like activities of macrophages via inflammasome activation, whereas ENHO^−/−^ mice had less M1 macrophages. The inflammasome activation in the adropin-treated macrophages was attributed to increased mROS due to increased oxidative metabolism in the mitochondria. Thus, an important role of adropin in CRC progression was addressed in this study, and adropin could be differently exploited to be a target in different CRC stages.

Tumor cells usually rely on anaerobic glycolysis to supply energy for rapid growth [29]. Hence, the decrease in adropin makes tumor cells use less mitochondrial energy. This phenomenon is definitely beneficial to tumor cell growth. However, increased adropin expression was identified in the macrophages in the late CRC stages (Dukes C-D), suggesting a metabolic shift in TAMs. In general, M1 macrophages preferentially use glycolysis, whereas M2 macrophages use fatty acid oxidation [30]. Thus, increased adropin in TAMs would help the pro-tumor effects of macrophages. Whether the intervention of ENHO in TAMs has the capacity to inhibit CRC metastasis and recurrence needs further exploration.

Notably, adropin had dual effects on macrophages. The macrophages had increased glucose oxidation after treatment with low-dose adropin (< 100 ng/mL), but fatty acid oxidation was only enhanced under the stimulation of high-dose adropin (> 100 ng/mL). The normal plasma adropin concentration in humans is 1–10 ng/mL [31]. For macrophages in the tumor microenvironment, the adropin concentration produced by adjacent cancer cells may be low. However, the adropin produced by the macrophages themselves possibly shapes the local microenvironment at high concentration. Thus, TAMs enhance the activity of M2-like cells by autonomous adropin to promote tumor invasion and metastasis. Additional studies are expected to elucidate the molecular mechanisms of how high-concentration adropin increases PPARγ and CPT1α expression in macrophages.

When MC38 cells were overexpressed with adropin and injected into mice, more CD8^+^ T cells were recruited into tumor tissues. We could not discriminate whether the CD8^+^ T cell activation was due to the direct effect of adropin or the indirect stimulation of adropin-impacted macrophages. On the one hand, how adropin directly affects effector T cell function still remains unknown. On the other hand, the loss of ENHO in individuals is associated with the loss of regulatory T cells [32] and leads to autoimmune diseases. Therefore, the effect of ENHO overexpression in carcinoma cells on the shaping of local adaptive immune function needs to be evaluated comprehensively.

## Conclusions

In conclusion, decreased adropin in carcinoma cells was involved in CRC progression. Low-dose adropin (< 100 ng/mL) could promote the induction of M1-like macrophages via increased glucose oxidation in the mitochondria, but high-dose adropin (> 100 ng/mL) induced M2-like macrophages *via* fatty acid oxidation. Considering that adropin is affected by exercise and diet, a healthy lifestyle can indeed inhibit tumor occurrence via enhancing immune surveillance.

### Electronic supplementary material

Below is the link to the electronic supplementary material.

**Supplementary table 1**-Clinical characteristics of 68 colorectal cancer patients. **Supplementary table 2**-Clinical characteristics of colorectal cancer patients with metastasis.

**Supplementary figure 1**. Phenotypic markers of macrophages with the treatment of adropin detected by flow cytometry. **Supplementary figure 2**. IL-1β and ROS production by macrophages with the treatment of adropin detected by flow cytometry. **Supplementary figure 3**. IL-1β and mROS production by macrophages with the treatment of higher-dose adropin detected by flow cytometry. **Supplementary figure 4**. Representative results of cell markers on macrophages or CD8+T cells detected by flow cytometry. (A) Macrophages in spleens of WT or KO mice. (B) M1 or M2 cells in WT or KO mice. (C) CD86 and CD206 on WT or KO macrophages treated with LPS/IFN-γ or IL-4. (D) NKG2D, CD69, IFN-γ or granzyme B (GZB) of CD8+T cells as cocultured with WT or KO macrophages.

Supplementary Material 3

## Data Availability

The datasets used and/or analysed during the current study are available from the corresponding author on reasonable request. The data that support the findings of this study are available from the corresponding author upon reasonable request. ENHO and GPR19 gene expression, immune infiltrates and related clinical information were from TCGA (https://portal.Gdc.Cancer.gov).
