# Correction: Low-dose adropin stimulates inflammasome activation of macrophage via mitochondrial ROS involved in colorectal cancer progression

**DOI:** 10.1186/s12885-023-11611-w

**Published:** 2023-11-10

**Authors:** Linghui Jia, Liting Liao, Yongshuai Jiang, Xiangyu Hu, Guotao Lu, Weiming Xiao, Weijuan Gong, Xiaoqin Jia

**Affiliations:** 1 Department of Basic Medicine, School of Medicine, Yangzhou University, Yangzhou, 225001 P. R. China; 2 Department of Gastroenterology, The Affiliated Hospital of Yangzhou University, Yangzhou, 225001 P. R. China; 3 Department of General Surgery, The Affiliated Hospital of Yangzhou University, Yangzhou, 225001 P. R. China; 4 Jiangsu Key Laboratory of Integrated Traditional Chinese and Western Medicine for Prevention and Treatment of Senile Diseases, Yangzhou, 225001 P. R. China; 5 Jiangsu Key Laboratory of Zoonosis, Yangzhou, 225001 P. R. China

**Correction: BMC Cancer 23, 1042 (2023)**

10.1186/s12885-023-11519-5

Following publication of the original article [1], the authors identified an error in the author list. The author Xiaoqin Jia was erroneously listed twice. This has now been corrected.

The author group has been updated above and the original article [1] has been corrected.
