# Supplementary material for: Low-dose adropin stimulates inflammasome activation of macrophage via mitochondrial ROS involved in colorectal cancer progression
Source: BMC Cancer. 2023 Oct 30;23:1042. doi: 10.1186/s12885-023-11519-5 (PMC10614368; doi:10.1186/s12885-023-11519-5)
Supplement: Supplementary file 1 — Supplementary table 1-Clinical characteristics of 68 colorectal cancer patients. Supplementary table 2-Clinical characteristics of colorectal cancer patients with metastasis. [file 12885_2023_11519_MOESM1_ESM.docx]

|  | **Supplementary table 1-**Clinical characteristics of 68 colorectal cancer patients | | | | | | | | |
| --- | --- | --- | --- | --- | --- | --- | --- | --- | --- |
| Characteristics | | Number | Dukes | | | | | χ2 | P |
|  |  |  | A-B | | | C | D |  |  |
| Age | |  |  | | |  |  | 2.379 | 0.304 |
| ＜60 | | 27 | 17 | | | 5 | 5 |  |  |
| ≥60 | | 41 | 18 | | | 12 | 11 |  |  |
| Gender | |  |  | | |  |  | 2.121 | 0.346 |
| Male | | 43 | 25 | | | 9 | 9 |  |  |
| Female | | 25 | 10 | | | 8 | 7 |  |  |
| Site | |  |  | | |  |  | 0.378 | 0.828 |
| Left | | 48 | 24 | | | 13 | 11 |  |  |
| Right | | 20 | 11 | | | 4 | 5 |  |  |
| Invasion depth | |  |  | | |  |  | 6.674 | 0.039 |
| <serosa | | 20 | 15 | | | 2 | 3 |  |  |
| ≥serosa | | 48 | 20 | | | 15 | 13 |  |  |
| Lymph node metastasis | | | | | | |  | 68 | <0.0001 |
| No | | 52 | 35 | | | 17 | 0 |  |  |
| Yes | | 16 | 0 | | | 0 | 16 |  |  |
| Distant metastasis | |  |  | | |  |  | 68 | <0.0001 |
| No | | 52 | 35 | | | 17 | 0 |  |  |
| Yes | | 16 | 0 | | | 0 | 16 |  |  |
| Differentiation | |  |  | | |  |  | 13.327 | 0.01 |
| well | | 22 | 9 | | | 3 | 10 |  |  |
| Moderate | | 45 | 26 | | | 14 | 5 |  |  |
| poor | | 1 | 0 | | | 0 | 1 |  |  |
| CEA | | 3.93（1.99，8.63） | | 3.85（2.07，5.84） | | 8.855(3.725,17.4075) | 3.44(1.78,6.86) | P_AB-C_ = 0.7675  P_A-D_ = 0.4848  P_C-D_ = 0.2332 | |
| CA199 | | 12.26(8.82，19.95) | 10.86（7.2，15.1） | | 14.1（10.4675，45.925） | | 13.1(8.785,20.8) | P_AB-C_ = 0.3813  P_A-D_ = 0.5652  P_C-D_ = 0.1819 | |

CEA, carcinoembryonic antigen; CA199, cancer antigen 199.

| **Supplementary table 2-**Clinical characteristics of colorectal cancer patients with metastasis | | | |
| --- | --- | --- | --- |
| Characteristics |  | Number | percentage（%） |
| Age |  |  |  |
|  | <60 | 0 | 0 |
|  | ≥60 | 4 | 100 |
| Gender |  |  |  |
|  | Male | 2 | 50 |
|  | Female | 2 | 50 |
| Differentiation |  |  |  |
|  | Poor | 4 | 100 |
|  | Well and Moderate | 0 | 0 |
| Primary focus site |  |  |  |
|  | Right hemicolon | 3 | 75 |
|  | Left hemicolon | 1 | 25 |
|  | Rectum | 0 | 0 |
| Depth |  |  |  |
|  | <serosa | 0 | 0 |
|  | ≥serosa | 4 | 100 |
| Lymph node metastasis |  |  |  |
|  | No | 1 | 25 |
|  | Yes | 3 | 75 |
| Transfer site |  |  |  |
|  | Liver | 4 | 100 |
| CEA（ug/l） |  |  |  |
|  | <200 | 1 | 25 |
|  | ≥200 | 1 | 25 |
|  | - | 2 | 50 |
