# Supplementary material for: Low-dose adropin stimulates inflammasome activation of macrophage via mitochondrial ROS involved in colorectal cancer progression
Source: BMC Cancer. 2023 Oct 30;23:1042. doi: 10.1186/s12885-023-11519-5 (PMC10614368; doi:10.1186/s12885-023-11519-5)
Supplement: Supplementary file 2 — Supplementary figure 1. Phenotypic markers of macrophages with the treatment of adropin detected by flow cytometry. Supplementary figure 2. IL-1β and ROS production by macrophages with the treatment of adropin detected by flow cytometry. Supplementary figure 3. IL-1β and mROS production by macrophages with the treatment of higher-dose adropin detected by flow cytometry. Supplementary figure 4. Representative results of cell markers on macrophages or CD8+T cells detected by flow cytometry. (A) Macrophages in spleens of WT or KO mice. (B) M1 or M2 cells in WT or KO mice. (C) CD86 and CD206 on WT or KO macrophages treated with LPS/IFN-γ or IL-4. (D) NKG2D, CD69, IFN-γ or granzyme B (GZB) of CD8+T cells as cocultured with WT or KO macrophages. [file 12885_2023_11519_MOESM2_ESM.docx]

Adropin (ng/ml)

0 10 30 100


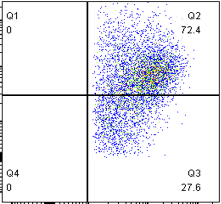

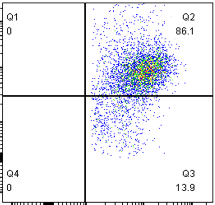

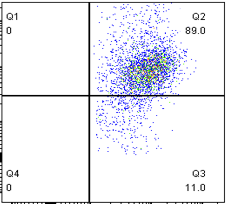

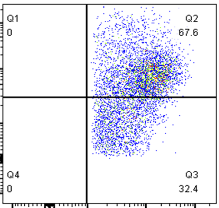


CD86


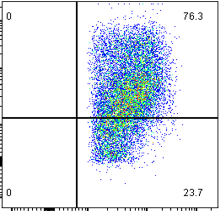

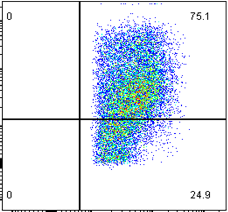

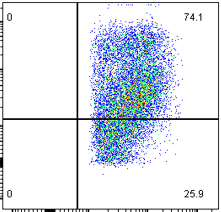

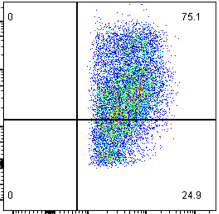


CD80


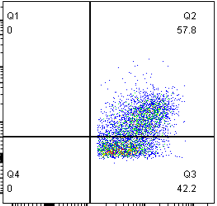

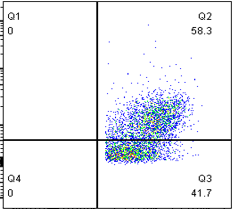

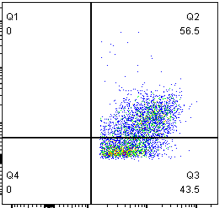

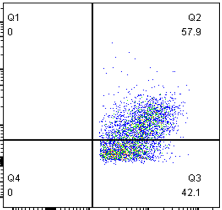


CD206

F4/80

Supplementary figure 1. Phenotypic markers of macrophages with the treatment of adropin detected by flow cytometry.

Adropin (ng/ml)

0 10 30 100


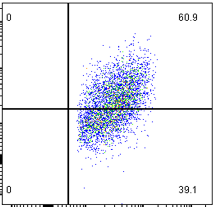

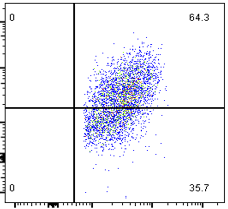

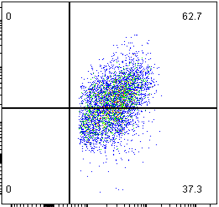

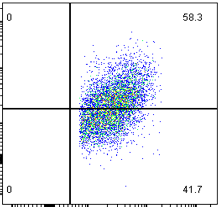


IL-1β


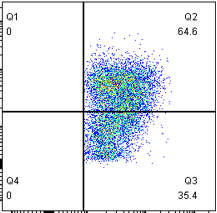

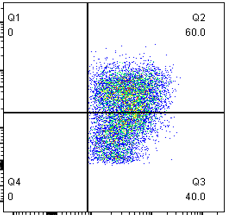

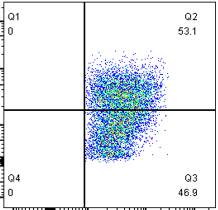

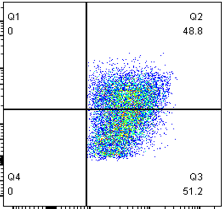


mROS


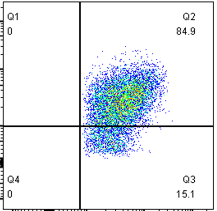

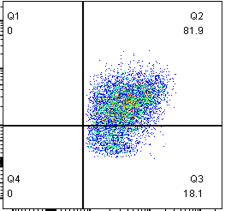

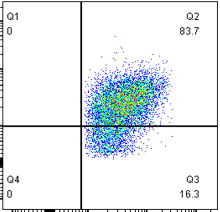

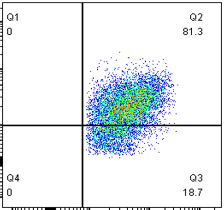


cROS

F4/80

Supplementary figure 2. IL-1β and ROS production by macrophages with the treatment of adropin detected by flow cytometry.


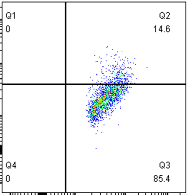

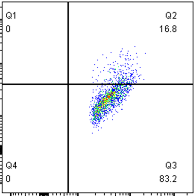

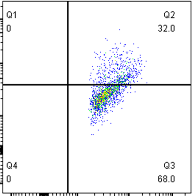

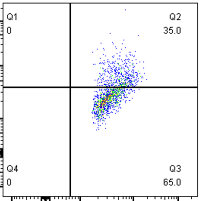

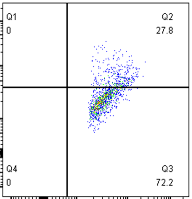

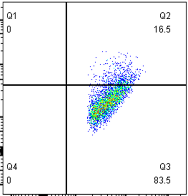


Adropin (ng/ml)

0 10 30 100 200 400

IL-1β


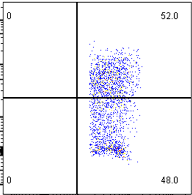

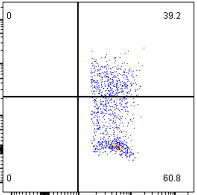

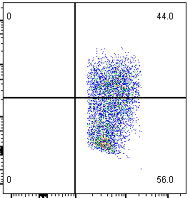

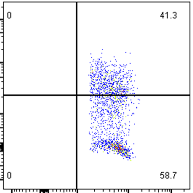

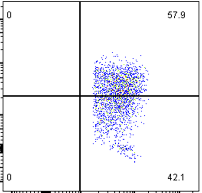

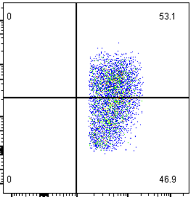


mROS

F4/80

Supplementary figure 3. IL-1β and mROS production by macrophages with the treatment of higher-dose adropin detected by flow cytometry.


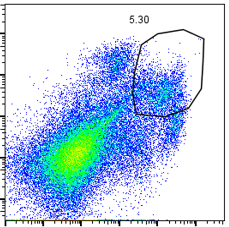

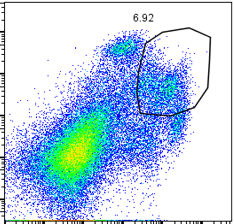


(A)

WT KO

F4/80

CD11b


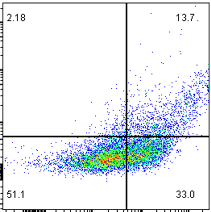

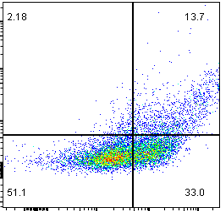

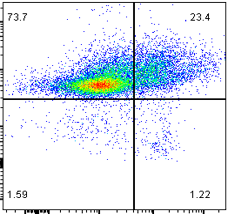

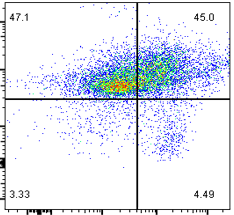


WT KO

(B)

WT KO

CD206

CD16/32

F4/80

F4/80


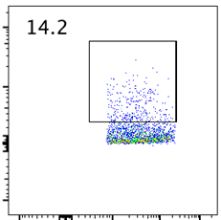

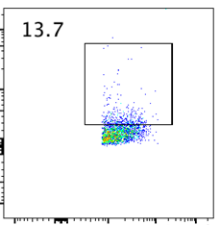

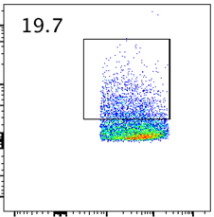

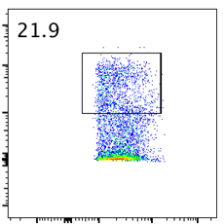

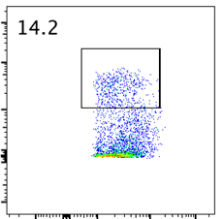

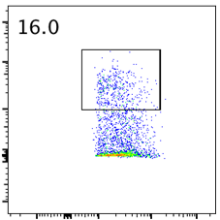

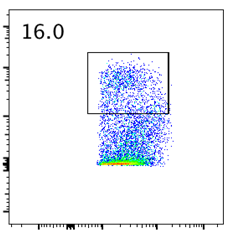


WT KO WT+Stimu KO+Stimu

(C)

CD86


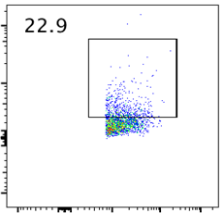


CD206

MAC^WT^ MAC^KO^  MAC^WT^ MAC^KO^

(D)

F4/80


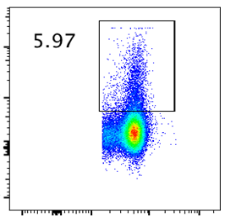

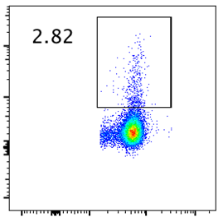

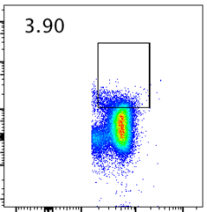

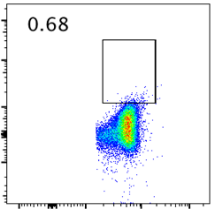

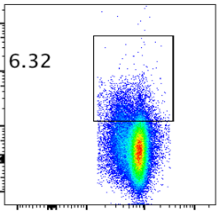

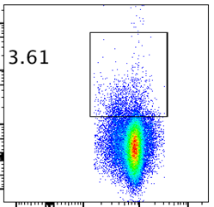

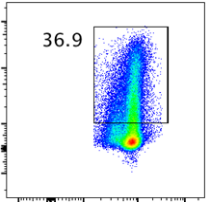

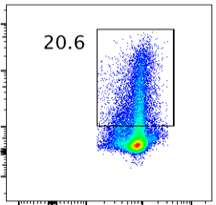


CD69

NKG2D

IFN-γ

GZB

CD8

CD8

Supplementary figure 4. Representative results of cell markers on macrophages or CD8^+^T cells detected by flow cytometry. (A) Macrophages in spleens of WT or KO mice. (B) M1 or M2 cells in WT or KO mice. (C) CD86 and CD206 on WT or KO macrophages treated with LPS/IFN-γ or IL-4. (D) NKG2D, CD69, IFN-γ or granzyme B (GZB) of CD8^+^T cells as cocultured with WT or KO macrophages.
