# Supplementary material for: Low-dose adropin stimulates inflammasome activation of macrophage via mitochondrial ROS involved in colorectal cancer progression
Source: BMC Cancer. 2023 Oct 30;23:1042. doi: 10.1186/s12885-023-11519-5 (PMC10614368; doi:10.1186/s12885-023-11519-5)
Supplement: Supplementary file 3 — Supplementary Material 3 [file 12885_2023_11519_MOESM3_ESM.docx]

（Protein molecular weight） 【The range of the crop】

3A

（H22 delete）H22 MC38 CMT93 CT26 NCM460 LS174T DLD Caco2 RKO HCT116 HT-29





Adropin （15kda）【10-25kda】





β-actin （45kda）【40-55Kda】

3B

(MC38 delete) pLV- MC38 pLV-ENHO- MC38


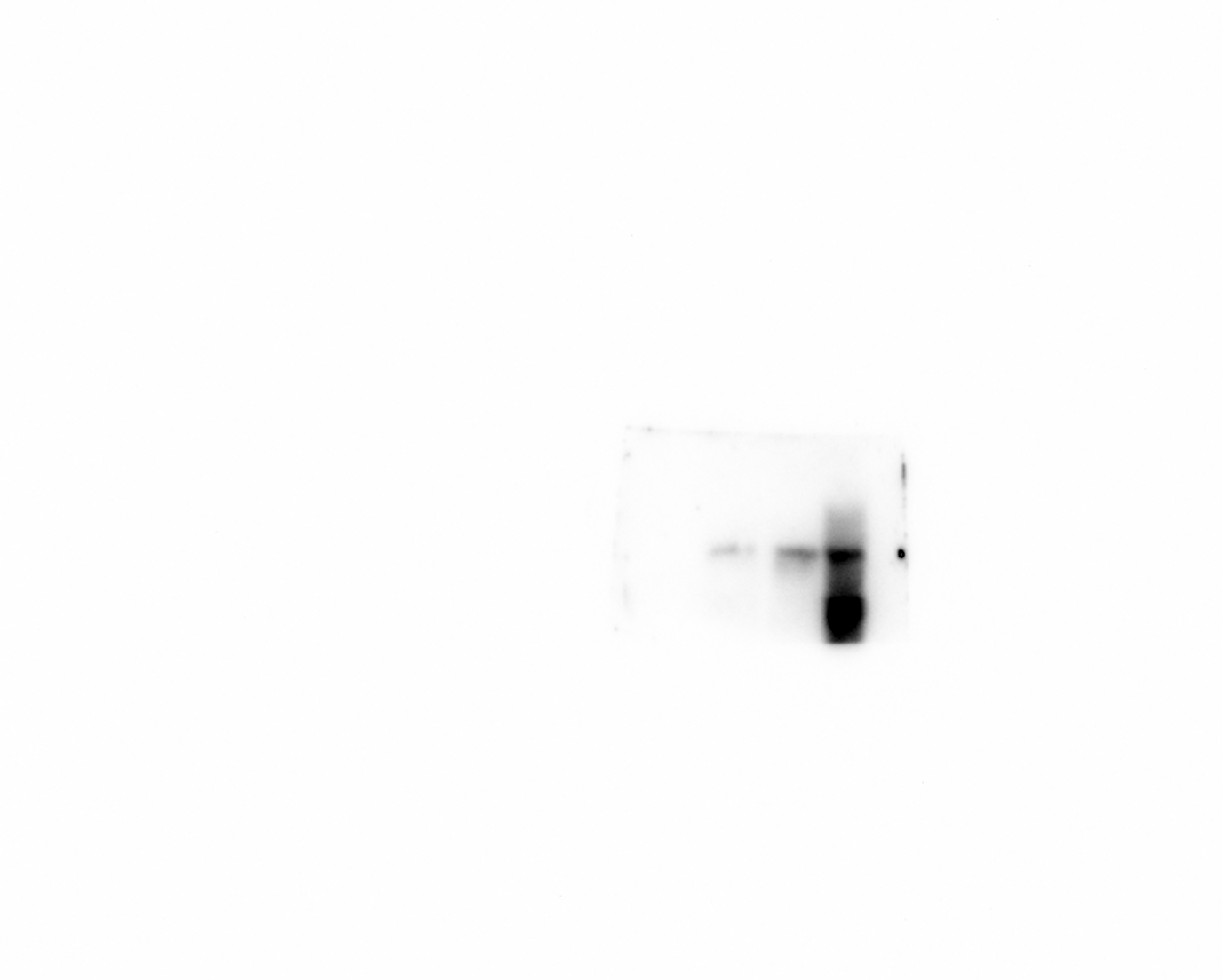


Adropin （15kda）【10-25kda】


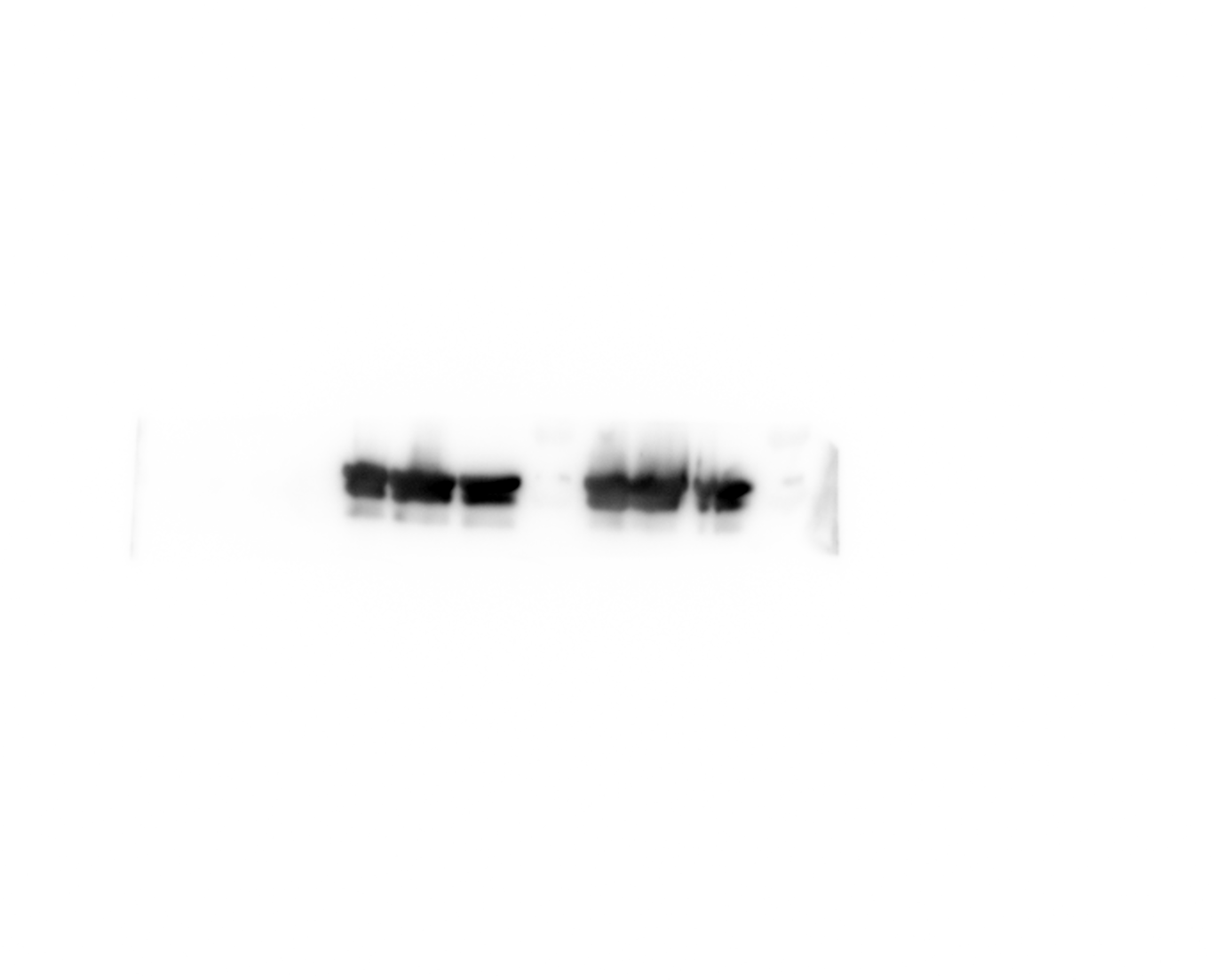


β-actin （45kda）【40-55Kda】

4D

Adropin (ng/ml) 0 10 30 100





NLRP3 （110kda） 【70-130kda】


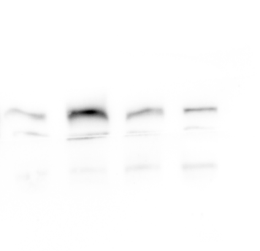


GSDMD （30,53kda） 【15-70kda】





c-caspase1 （20kda） 【10-55kda】





c-IL-1β （17kda） 【10-55kda】





β-actin （45kda） 【40-55kda】

4E

Adropin (ng/ml) 0 10 30 100





iNos (130kda) 【100-150Kda】





ARG1 （35kda）【25-55Kda】





β-actin （45kda）【40-55Kda】

5A

PBS LPS/IFN-γ IL-4 IL-10 TGF-β1





Adropin （15kda）【10-25kda】





β-actin （45kda）【40-55Kda】

5E

WT1 WT2 KO1 KO2





NLRP3 （110kda） 【70-130kda】





GSDMD （30,53kda） 【15-70kda】





c-IL-1β （17kda） 【10-40kda】





β-actin （45kda） 【40-55kda】


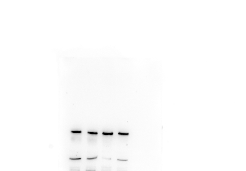


c-caspase1 （20kda） 【10-55kda】

5F

WT1 WT2 KO1 KO2





INOS (130kda) 【100-150Kda】


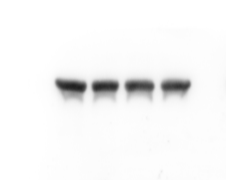


β-actin （45kda） 【40-55kda】





ARG1 （35kda） 【20-55kda】

6A

Adropin (ng/ml) 0 10 30 100 200 400





β-actin （45kda）【40-55Kda】





Cpt1α （86kda）【70-100Kda】





HK2 （102kda）【70-130Kda】





Glut1 （45kda）【35-55Kda】





PPARγ （57，53kda）【40-70kda】





C/EBPβ （35kda）【25-40kda】

6B

Adropin (ng/ml) 0 10 30 100 200 400





mtor （289kda） 【200kda-】





AKT （60kda）【40-70kda】





P-mtor （289kda） 【200kda-】





P-AKT （60kda） 【40-70kda】





β-actin （45kda）【40-55kda】





AMPK （62kda） 【55-70kda】


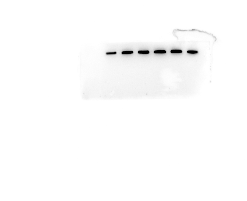


P-AMPK （62kda）【35-70kda】

6C

WT1 WT2 KO1 KO2





C/EBPβ （35kda） 【25-40kda】





β-actin （45kda） 【40-55kda】





CPT1α （102kda） 【70-130kda】





GLUT1 （45kda）【35-70kda】





HK2 （102kda）【70-130kda】





PPAR-γ （53kda）【40-70kda】

6D

WT1 WT2 KO1 KO2





AKT （60kda）【40-100kda】





AMPK （62kda） 【55-100kda】





β-actin （45kda） 【40-55kda】





mtor （289kda）【200kda-】





P-AKT （60kda）【40-100kda】





P-AMPK （62kda）【55kda】





P-mtor （289kda）【200kda-】

6E

NLRP3 （110kda） 【70-130kda】







GSDMD （53,30kda） 【15-70kda】





β-actin （45kda） 【40-70kda】
